# Monocytic C-C chemokine receptor 5 expression increases in in vitro intermittent hypoxia condition and in severe obstructive sleep apnea patients

**DOI:** 10.1007/s11325-019-01797-4

**Published:** 2019-02-18

**Authors:** Li-Pang Chuang, Ning-Hung Chen, Shih-Wei Lin, Han-Chung Hu, Kuo-Chin Kao, Li-Fu Li, Cheng-Ta Yang, Chung-Chi Huang, Jong-Hwei S. Pang

**Affiliations:** 1 Sleep Center, Department of Pulmonary and Critical Care Medicine, Chang Gung Memorial Hospital, Taoyuan City, Taiwan; 2 School of Medicine, College of Medicine, Chang Gung University, Taoyuan City, Taiwan; 3 Graduate Institute of Clinical Medical Sciences, College of Medicine, Chang Gung University, Taoyuan City, Taiwan; 4 Department of Physical Medicine and Rehabilitation, Chang Gung Memorial Hospital, Linkou, Taoyuan City, Taiwan

**Keywords:** Intermittent hypoxia, Monocyte, Chemokine receptor, Chemotaxis, Obstructive sleep apnea

## Abstract

**Purpose:**

Obstructive sleep apnea (OSA) patients have higher risk of cardiovascular disease. C-C chemokine receptor 5 (CCR5), as an important receptor for monocyte recruitment and the initiation of atherosclerosis, was studied under intermittent hypoxia and in OSA patients.

**Methods:**

The expression and function of CCR5 regulated by intermittent hypoxia in monocytic THP-1 cells were investigated in an in vitro intermittent hypoxia culture system. The expression levels of protein and mRNA were analyzed by western blot and RT/real-time PCR analysis. Cell adhesion assay and transwell filter migration assay were carried out to investigate the adhesion and chemotaxis of monocytes. In addition, the mRNA expression of CCR5 in monocytes isolated from peripheral blood of 72 adults was analyzed.

**Results:**

Intermittent hypoxia upregulated the expression of CCR5 in THP-1 cells and enhanced the adhesion and chemotaxis of monocytes to vascular endothelial cells mediated by RANTES. The CCR5 expression induced by intermittent hypoxia was inhibited by inhibitor for p42/44 MAPK. Besides, the expression of CCR5 in monocytes increased along the AHI value especially in severe OSA patients that was statistically significant compared with mild and moderate OSA groups.

**Conclusions:**

This study demonstrated the increased monocytic CCR5 gene expression in patients with severe OSA. Intermittent hypoxia, the characteristic of OSA, induced monocytic CCR5 gene expression and the enhanced RANTES-mediated chemotaxis and adhesion through p42/44 MAPK signal pathways.

## Introduction

Obstructive sleep apnea (OSA), which affects more than 5% of the adult population, is an increasing prevalent disease [1]. It is characterized by repetitive episodes of complete or partial upper airway obstruction when asleep, resulting in subsequent arousals [2]. The published literatures show that OSA patients have an increased risk of cardiovascular events, such as hypertension, myocardial infarction, heart failure, nocturnal dysrhythmias, and pulmonary hypertension [3]. It has been reported that exaggerated negative pressure in thorax, hypercapnia, intermittent hypoxia, and surges of sympathetic activity might contribute to these cardiovascular diseases mediated through the endothelial dysfunction [4].

Among these cardiovascular diseases, coronary heart disease is the result of the accumulation of atheromatous plaques within the coronary artery walls [5]. Some studies have showed the high prevalence of OSA among patient with coronary heart disease and high prevalence of coronary heart disease among patient with OSA [6, 7]. Intermittent hypoxia could activate the inflammatory reaction which is critically involved in OSA as demonstrated by both in vitro and in vivo studies [8]. The activation of leukocyte and endothelial cell and the adhesion of leukocyte to endothelium are known to result in the inflammation and the development of atherosclerosis [9]. The transmigration of circulating monocytes into vascular intimal space initially attracted by various chemokines secreted by vascular endothelial cells is an important tread in the progression of atherosclerosis [10].

Regulated upon activation normal T cell expressed and secreted (RANTES), an 8-kDa polypeptide of the C-C chemokine family, was a mighty chemotactic factor for monocytes and T lymphocytes [11]. RANTES was expressed highly in atheroma, and higher plasma RANTES levels were related to the extent of carotid atherosclerosis and high-risk plaques [12]. C-C chemokine receptor type 5 (CCR5), one of the receptors of RANTES, is a G protein–coupled receptor that belongs to the beta chemokine receptor family of integral membrane proteins [13]. Blocking RANTES/CCR5 signaling with antagonist in vivo influences the development of atherosclerotic lesions [14].

Although one study revealed that inhibition of RANTES attenuated intermittent hypoxia (IH)–evoked inflammatory preatherosclerotic remodeling and some literatures demonstrated that hypoxia can induce CCR5 expression [15–17], there are still puzzles in understanding whether “intermittent hypoxia” can activate monocyte to express more CCR5, which causes subsequent atherosclerosis development. Also, no currently published literature has mentioned about the changes of CCR5 expression in monocyte from OSA patients. In the present study, we therefore investigated how intermittent hypoxia affects the regulation of CCR5 expression and related signal transduction pathways in monocytic THP-1 cells. The CCR5 expression was also examined in monocytes isolated from OSA patients.

## Materials and methods

### Materials

As described in detail previously [18], THP-1 cells, the human monocytic leukemia cells purchased from ATCC, were cultured in RPMI 1640 medium containing 10% FBS and antibiotics. Vascular endothelial cells isolated from human umbilical vein (HUVECs) were purchased from BCRC (Taiwan, ROC) and grown in EGM provided by Clonetics Inc. (MD, USA). Monoclonal anti-CCR5 was purchased from Epitomics Inc. (CA, USA). Recombinant RANTES was obtained from R&D Systems (MN, USA).

### Normoxia and intermittent hypoxia culture conditions

As described in detail previously [18], 1 × 10^6^ cells/ml human blood monocytes or THP-1 cells were cultured in RPMI 1640 medium. Experimental condition was set up in a modified Hyper-Hypo Oxygen System developed by NexBioxy. (Taipei, Taiwan). Cells were cultured in normoxia or intermittent hypoxia following the detailed conditions described in previous studies [18, 19]. Cells were maintained in standard culture incubator for another 18 h and then harvested for following experiments.

### RNA isolation and RT/real-time PCR

Total RNA was extracted from THP-1 cells using TRIzol™ reagent following manufacturer’s protocol [20]. Total cellular RNA was applied to synthesize cDNA by M-MLV reverse transcriptase (USB Corporation, OH, USA). Primers used for PCR were forward primer for CCR5, 5′-GCTGTGTTTGCGTCTCTCCCAGGA-3′, reverse primer for CCR5, 5′-CTCACAGCCCTGTGCCTCTTCTTC-3′; forward primer for GAPDH, 5′-GACCTGACCTGCCGTCTA-3′, reverse primer for GAPDH, 5′-AGGAGTGGGTGTCGCTGT-3′. Real-time PCR was performed following the protocol described in our previous study [18].

### Western blot analysis

Proteins prepared from cell membrane were used for western blot analysis, and monoclonal antibody against CCR5 was applied as described previously [18]. The band density was measured by the 1D Digital Analysis Software, Kodak Digital Science™ (Eastman Kodak, NY, USA). Normalized data were expressed as 100% in normoxia control group.

### Migration assay and cell adhesion assay

The chemotaxis assay used transwell inserts with 8 μm membrane pores (Costar, Cambridge, MA), with lower chamber containing 30 ng/ml RANTES and monocytes set to migrate for 1 h as described previously [18].

### Clinical patients

By screening patients of possible OSA in our sleep center, a total of 72 adults (> 20 years old) patients were included. The exclusion criteria were as follows: chronic or recently diagnosed inflammatory or infectious condition such as invasive surgical/medical/dental procedure, trauma and asthma; the use of antibiotics or anti-inflammatory drugs within recent 1 month; and the existence of hypertension, coronary heart disease, hyperlipidemia, cerebrovascular disease, diabetes, or renal disease. This study was agreed by the Institutional Review Board of Chang Gung Memorial Hospital (No.100-3166B), and informed consent in written form was acquired from every patient before the study.

### Polysomnography

Standard overnight polysomnography (PSG) was performed using the Siesta Physiological Monitoring System (Abbotsford, Australia) in Chang Gung Memorial Hospital sleep center. Respiratory movements of the abdomen and chest were detected by inductive plethysmography bands. Air flow from mouth and nose was measured by thermistors. The oxygen saturation (SpO_2_) from artery was monitored transcutaneously with pulse oximetry which is hooked on the fingertip. Apnea events were defined as described in our previous study [18]. The severity of OSA was estimated by AHI (normal: AHI ≦ 5; mild: 5 < AHI ≦ 15; moderate: 15 < AHI ≦ 30; and severe: AHI > 30). The oxygen desaturation index (ODI) was calculated by dividing the number of the arterial oxygen level drops more than 3% from baseline by the hours of estimated total sleep time.

### Peripheral blood drawling and monocyte isolation

Venous blood was obtained from patients at 6 A.M. and the next morning after the PSG study under fasting condition in supine position. Mononuclear cell of peripheral blood was isolated following the protocols described in previous studies [18, 21].

### Statistical analysis

Non-parametric test using Wilcoxon signed-rank test was used for analysis of the difference of mRNA or protein expression. One-way analysis of variance (ANOVA) was applied for analysis of the difference of parametric data of three or more groups. Correlations and multiple regression analyses were applied to verify the relationship between CCR5 expression with AHI, ODI, BMI, and age. The statistical analysis was carried out with the software from SPSS (Chicago, USA). The data was expressed as mean ± SEM, and a *p* value ≦ 0.05 was used to show statistical significance.

## Results

### CCR5 gene expression was upregulated by intermittent hypoxia

**Fig. 1:**
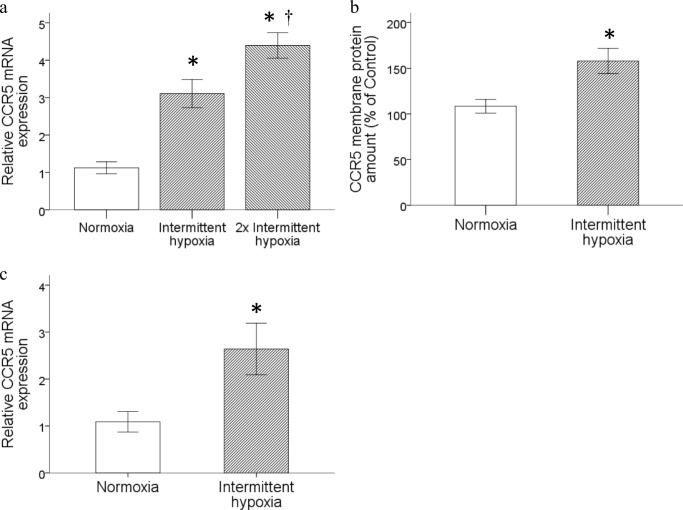
Intermittent hypoxia enhanced CCR5 gene expression in monocytic THP-1 cells. Monocytic THP-1 cells were treated with normoxia or intermittent hypoxia as described in the Material and methods section. **a** RNA was isolated for the analysis of CCR5 gene expression by RT/real-time PCR. **b** Membrane proteins were prepared for western blot analysis. **c** Human peripheral monocytes were treated with the same conditions as in (**a**) and total RNA was isolated for the analysis of CCR5 gene expression by RT/real-time PCR. (Data are presented as mean ± SEM, *p < 0.05 vs. Normoxia, ^†^*p* < 0.05 vs. Intermittent hypoxia)

The consequence of intermittent hypoxia on the mRNA and protein levels of CCR5 expression in monocytic THP-1 cells was studied. Monocytic THP-1 cells were treated by intermittent hypoxia or normoxia as described in the Materials and methods section. Intermittent hypoxia upregulated the CCR5 mRNA expression in monocytic THP-1 cells, and a more significant increase could be induced under a condition with a double dose of intermittent hypoxia (Fig. 1a). The result obtained by western blot analysis further demonstrated that the membrane CCR5 proteins isolated from THP-1 cells were significantly amplified by intermittent hypoxia (Fig. 1b). The upregulation of CCR5 mRNA expression by intermittent hypoxia was also demonstrated in human monocytes isolated from peripheral blood under the same culture condition (Fig. 1c).

### Intermittent hypoxia increased chemotaxis of monocytic THP-1 cells toward RANTES

**Fig. 2:**
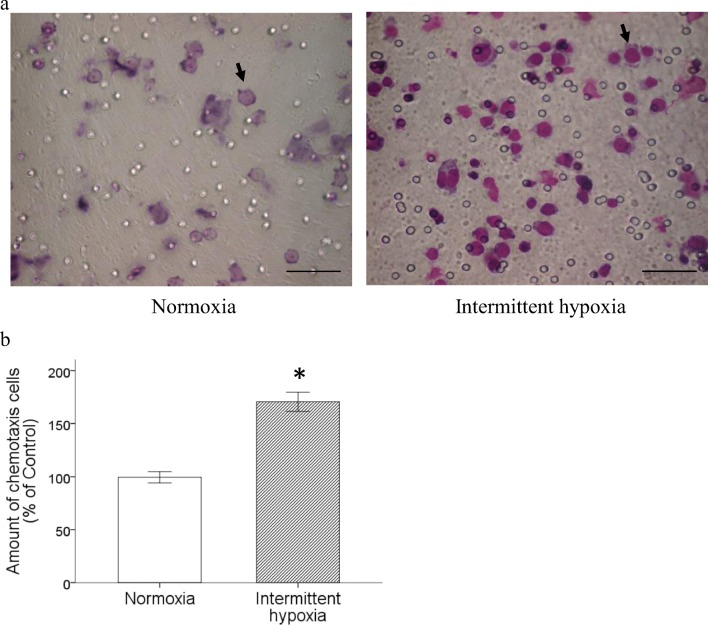
Intermittent hypoxia enhanced RANTES-induced chemotaxis of monocytic THP-1 cells. Monocytic THP-1 cells were treated with normoxia or intermittent hypoxia as described, and RANTES-mediated chemotaxis were processed. **a** Photos represented for normoxia-treated or intermittent hypoxia-treated monocytic THP-1 cells that migrated toward lower chamber through the transwell filter. Chemotaxis cells were indicated by black arrow. Scale bar = 100 μm. **b** Statistical results from three experiments showed significantly enhance the chemotaxis toward RANTES. (Data are presented as mean ± SEM; *p < 0.05 vs. Normoxia)

The different chemotaxic potentiality of THP-1 cells toward RANTES was analyzed by transwell migration assay using cells incubated under the condition of intermittent hypoxia or normoxia as described in Materials and methods. The result showed that intermittent hypoxia markedly promoted the chemotaxic ability of monocytic THP-1 cells stimulated by RANTES (Fig. 2a and b).

### Intermittent hypoxia enhanced RANTES-stimulated adhesion of monocytic THP-1 cells to vascular endothelial cells

**Fig. 3:**
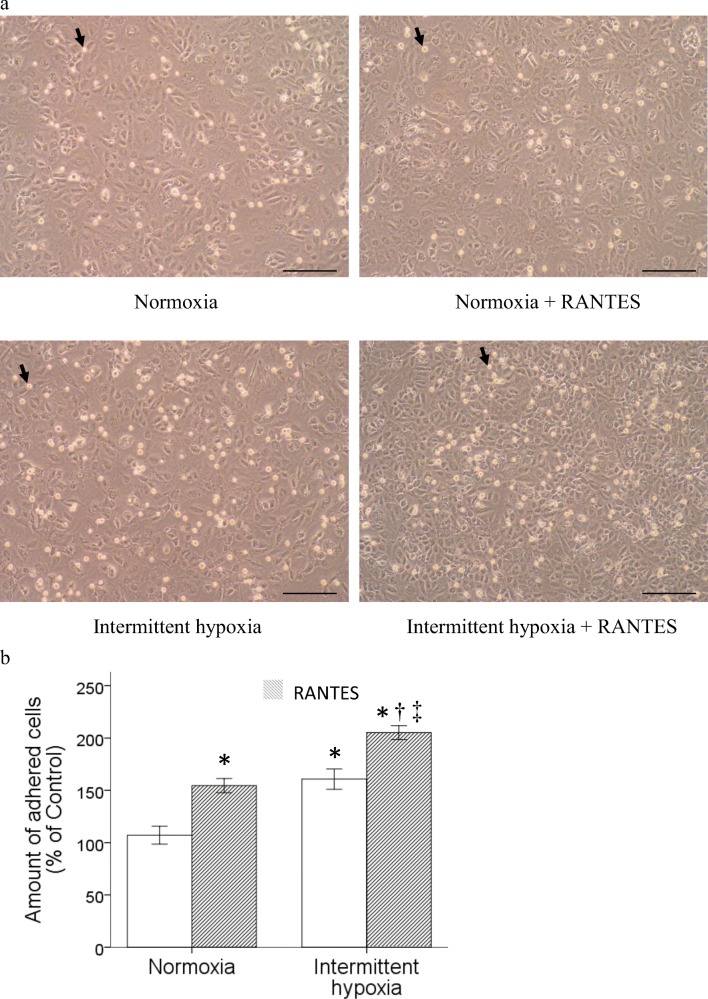
Intermittent hypoxia enhanced the RANTES-stimulated adhesion of monocytic THP-1 cells to vascular endothelial cells. Pretreated monocytic THP-1 cells with normoxia or intermittent hypoxia were activated by 30 ng/ml RANTES for another 18 h, and then processed for adhesion assay. **a** Photos represented for monocytic THP-1 cells after cell adhesion assay. Black arrow indicated the adhered cells. Scale bar = 100 μm. (Normoxia: without any treatment, Normoxia + RANTES: with RANTES stimulation only, Intermittent hypoxia: with intermittent hypoxia pretreatment only, Intermittent hypoxia + RANTES: with intermittent hypoxia pretreatment and RANYES stimulation.) **b** Statistical results from three independent experiments showed intermittent hypoxia treatment synergistically promoted the adhesive activity of monocytic THP-1 cells. (Data are presented as mean ± SEM; *p < 0.05 vs. Normoxia, ^†^*p* < 0.05 vs. Normoxia + RANTES, ^‡^*p* < 0.05 vs Intermittent hypoxia)

Monocytic THP-1 cells were treated with normoxia or intermittent hypoxia as described in the previous section and used for the assay of RANTES-stimulated adhesion to vascular endothelial cells. Treatment with intermittent hypoxia alone or 30 ng/ml RANTES amplified the adhesion of monocytic THP-1 cells to the vascular endothelial monolayer. Interestingly, treatment with the combined RANTES and intermittent hypoxia synergistically enhanced the adhesion ability of monocytic THP-1 cells (Fig. 3a and b).

### Antagonist of P44/42 suppressed the intermittent hypoxia-induced CCR5 expression

**Fig. 4:**
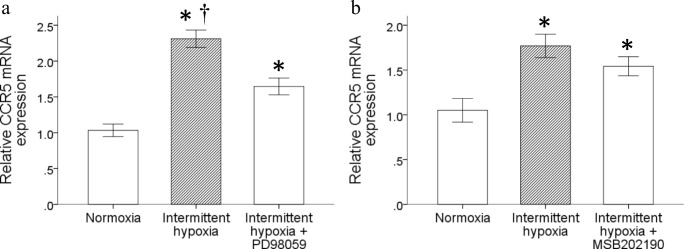
P44/42 antagonist inhibited the increase of CCR5 expression induced by intermittent hypoxia. Monocytic THP-1 cells were pretreated for 1 h with **a** 10 μM PD98059 or **b** 20 μM MSB202190 to inhibit p44/42 or p38 MAPK pathway respectively, followed by treatment with intermittent hypoxia, then cultured in normal incubator for 18 h. RNA was isolated for the analysis of CCR5 mRNA expression by RT/real-time PCR. (Data are presented as mean ± SEM, *p < 0.05 vs. Normoxia, ^†^*p* < 0.05 vs. Intermittent hypoxia + PD98059)

We then investigated the signal pathway responsible for the upregulation of CCR5 expression in monocytes by intermittent hypoxia. Results showed that pretreatment with 10 μM PD98059 but not 20 μM SB202190 for 1 h downregulated the intermittent hypoxia-induced CCR5 gene expression (Fig. 4a and b, respectively). It demonstrated that the activation of p44/42 signaling pathway was needed for the upregulated CCR5 gene expression in monocytes triggered by intermittent hypoxia.

### Increased CCR5 mRNA expression in monocytes of patients with OSA

**Fig. 5:**
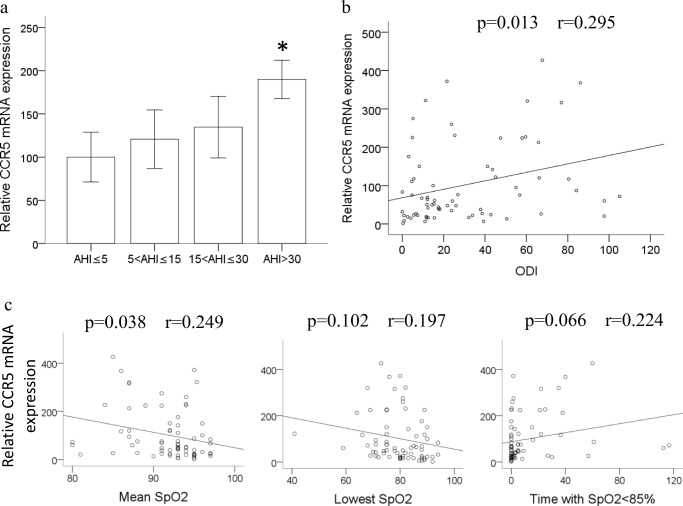
CCR5 mRNA expression increased in monocytes of OSA patients. **a** The CCR5 mRNA expression of 72 patients from four groups with different OSA severity was analyzed by RT/real-time PCR. (Data are presented as mean ± SEM, *p < 0.05 vs. AHI ≤ 5.) **b** Linear regression demonstrated the positive correlation between ODI and CCR5 mRNA expression levels in monocytes (*p* = 0.013, *r* = 0.295). **c** Linear regression demonstrated the positive correlation between CCR5 mRNA expression levels in monocytes and mean SpO_2_ (*p* = 0.038, *r* = 0.249), but not lowest SpO_2_ or time with SpO_2_ < 85%

Seventy-two patients who participated in our study were separated into four groups in accordance with the OSA severity. Table 1 is the basic demographic data of OSA patients. No statistical significance was founded over age among these groups. The body mass index (BMI) and the variables of PSG including mean SpO_2_, lowest SpO_2_, time with SpO_2_ < 85%, and ODI indicated statistical significance among four different groups. The same investigation of the expression of CCR5 mRNA by RT/real-time PCR was performed using monocytes isolated from the venous blood of these OSA patients. The CCR5 mRNA gene expression was demonstrated to be elevated along the OSA severity especially in the AHI > 30 group which was statistically significant comparing with the normal group (AHI ≦ 5) (Fig. 5a). Besides, results in Fig. 5b also revealed the positive association between ODI and the levels of CCR5 mRNA expression (*p* = 0.013, *r* = 0.295). In Fig. 5c, CCR5 mRNA expression has positive correlation with mean SpO_2_ (*p* = 0.038, *r* = 0.249), but not lowest SpO_2_ or time with SpO_2_ < 85%. Only AHI and ODI were demonstrated to associate positively with monocytic CCR5 expression (*p* = 0.006 and *p* = 0.031 respectively), but not age (*p* = 0.780) or BMI (*p* = 0.113) after multiple regression analyses.

**Table 1:** Demographic data and polysomnography parameters

	Severity				
	AHI ≦ 5	5 < AHI ≦ 15	15 < AHI ≦ 30	AHI > 30	
No. of subjects; (male)	10 (10)	17 (15)	16 (14)	29 (27)	
Age, years	42.6 ± 3.7	43.8 ± 3.0	41.4 ± 2.2	43.5 ± 2.0	*p* = 0.913
BMI, kg/m^2^	24.2 ± 0.9	24.5 ± 1.0	26.3 ± 0.8	28.6 ± 0.8^abc^	*p* = 0.001
AHI, events/hour	2.0 ± 0.5	10.3 ± 0.8^a^	20.8 ± 1.0^ab^	57.5 ± 3.4^abc^	*p* < 0.001
Sleep efficiency, %	75.0 ± 7.0	73.1 ± 3.3	71.5 ± 3.1	67.4 ± 3.1	*p* = 0.510
ODI, events/hour	1.6 ± 0.6	9.6 ± 1.0^a^	18.2 ± 1.5^ab^	57.7 ± 4.3^abc^	*p* < 0.001
Mean SpO_2_, %	94.3 ± 1.0	94.0 ± 0.5	93.5 ± 0.6	88.7 ± 0.8^abc^	*p* < 0.001
Lowest SpO_2_, %	89.0 ± 2.2	85.4 ± 0.9	79.3 ± 1.5^ab^	73.2 ± 1.6^abc^	*p* < 0.001
Time with SpO_2_ < 85%, minutes	0.6 ± 0.6	0.5 ± 0.2	2.6 ± 0.7^b^	27.5 ± 5.8^abc^	*p* < 0.001

*BMI*, body mass index; *AHI*, apnea-hypopnea index; *ODI*, 4% oxygen desaturation index; *SpO*_*2*_, oxygen saturation. (Data are presented as mean ± SE; ^a^*p* < 0.05 vs. AHI ≦ 5, ^b^*p* < 0.05 vs. 5 < AHI ≦ 15, and ^c^*p* < 0.05 vs. 15 < AHI ≦ 30)

## Discussion

We demonstrated in the present study that intermittent hypoxia can stimulate the monocytes to actively express CCR5 at both the membrane protein levels and mRNA, which subsequently increased the migratory ability of monocytes toward RANTES and adhesion to endothelial cell. Besides, the p44/42 MAPK pathway was demonstrated to contribute to the activation of monocytes by intermittent hypoxia. Furthermore, increased monocytic CCR5 expression was found in severe OSA patients.

It has been demonstrated that some chemokines and their receptors are in charge of the adhesion, transendothelial migration, and chemotaxis of monocytes which is important in the initiation of atherosclerosis [22]. Studies using ApoE-null mice combined with the deficient chemokine or its receptor have further confirmed their roles in the pathogenesis of atherosclerosis [23]. C-C chemokine receptor type 5 (CCR5), which belongs to the beta chemokine receptors family of integral membrane proteins, is expressed in peripheral blood leukocytes, including monocytes, macrophages, and T cells [13, 24]. It regulates leukocyte chemotaxis in inflammation and serves as a functional receptor for various inflammatory CC-chemokines [25]. Among these chemokines, RANTES immobilized on activated endothelium can trigger leukocyte transmigration, which is mediated by specialized roles of CCR5 [26]. Also, in an animal study, CCR5 plays a role in the recruitment and activation of leukocytes as well as of vascular cells, and the blockade of CCR5 by RANTES antagonist would prevent leukocyte migration into lesion [14]. In OSA patients, serum RANTES level was found to be independently associated with AHI after an acute cardiovascular event [27]. In this study, the upregulation of CCR5 expression in monocytes of severe OSA patient was confirmed. The earlier published data showed that the level of RANTES markedly higher in OSA patients might further augment the consequence of intermittent hypoxia on the adhesion and chemotaxis of monocytes toward endothelial cells.

The CCR5 gene expression has been reported to be induced by hypoxia in dendritic cells [28], and hypoxia-ischemic injury can enhance CCR5 gene expression localized to endothelium in rat brain [29]. Many studies have demonstrated that the expression of CCR5 is absolutely critical in human immunodeficiency virus (HIV)–positive patients and various CCR5 inhibitors have been developed to treat HIV disease [30]. In recent years, obstructive sleep apnea has been increasingly reported in HIV patients [31, 32]. This study was the first to show that in vitro intermittent hypoxia can upregulate the monocytic CCR5 expression at the protein levels and mRNA. Besides, we further examined the correlation between human monocytic CCR5 expression with oxygen parameters in PSG and revealed that only ODI and mean SpO_2_ have positive correlation with CCR5 gene expression but not lowest SpO_2_ or time with SpO_2_ < 85%. These results supported our point of view that the increased CCR5 expression correlates more with the frequency of hypoxemic episodes rather than the duration of the hypoxemic episodes, or the severity of hypoxemia.

The upregulation of monocytic CCR5 mRNA expression has been found to be mediated by the activation of some signal pathways such as p44/42 or p38 MAPK [33, 34]. The inhibition of p44/42 and p38 MAPK signal pathway in monocytes by PD98095 and MSB202190 respectively diminished the CCR5 gene expression enhanced by different stimulator [35, 36]. However, there is no reported investigation on the CCR5 gene expression pathway under hypoxia or intermittent hypoxia. In our study, the inhibition of p44/42 by PD98095 decreased the monocytic CCR5 gene expression stimulated by intermittent hypoxia. On the other hand, there was no inhibitory effect on the intermittent hypoxia-induced monocytic CCR5 expression by pretreatment with p38 MAPK inhibitor. Together, results revealed the activation of p44/42 was needed for the upregulated monocytic CCR5 expression triggered by intermittent hypoxia.

Some studies have revealed the underlying mechanism and possible pathway in intermittent hypoxia and atherosclerosis by in vivo and in vitro studies. The underlying mechanisms of intermittent hypoxia related to the atherosclerosis formation include inflammation, oxidative stress, platelet activation, cell apoptosis, vascular endothelial injury, insulin resistance, and neuroendocrine disorders [37]. Endothelial cell injury is an important mechanism of atherosclerosis, and chemotaxis plays the initial crucial role in that process [22]. We have found in our previous reports that the upregulated expression of MCP-1 and CCR2 in monocytes of patients with severe OSA and monocytic MCP-1 and CCR2 gene expression can be activated under intermittent hypoxia which subsequently promotes the adhesion and chemotaxis of monocytes [18, 20]. Combined together, this present study proved that intermittent hypoxia can induce CCR5 expression in monocytes, directly supporting the interpretation that intermittent hypoxia can enhance the chemotaxic ability of monocytes, which consequently results in more cardiovascular events in severe OSA patients. This is truly another new possible molecular mechanism that differs from other studies. In addition to atherosclerosis, pulmonary hypertension is frequently seen in patients with OSA, and the coexistence of pulmonary hypertension and OSA predicts a worse prognosis and higher morbidity [38]. It is interesting to find out that the marked CCR5 expression in the macrophages of the lungs from patients with pulmonary hypertension might represent a new therapeutic target to modulate the cell growth and arterial remodeling [39].

The novelty of our study is to prove the upregulated gene expression of monocytic CCR5 in OSA patient; however, there are some limits that can be discussed. We have excluded the potential confounders in this study that might interfere with the CCR5 expression such as ischemic heart disease. Although we tried to exclude the potential cause of systemic inflammation as much as we could, there are still myriad of possible causes of inflammation beyond we thought. It is a better design to test the inflammatory effect of intermittent hypoxia in the same patient group to eliminate the possible confounders. In addition, body weight is proved to be an important factor affecting OSA. It is difficult to enroll patients with high BMI and without any respiratory event during sleep. We separated recruited patients into four groups in accordance with the OSA diagnostic criteria; the ODI and AHI in 72 patients were noticeably different among these groups. Although the BMI was also significantly different among groups, the possible effect of BMI was excluded because only ODI and AHI have positive correlation with monocytic CCR5 expression after multiple regression analyses. Although OSA is obviously a chronic disorder, the night time events, such as intermittent hypoxia, may have acute effects contributing to this disease. According to the published paper by Dr. Tamaki, they found that just one-night hypoxic stress can activate the invasive function of monocytes in patients with OSA [40]. Also, our previous published literature comparing the serum MMP-9 expression in patients with OSA revealed the same phenomenon that just one-night events can increase MMP-9 expression after sleep [21]. It seems that some parts of OSA-related injury occur on a nightly basis. On the other hand, during event-free daytime, some injuries may have been recovered in somewhat degree, depending on the severity of injury itself and host repair ability [41]. Thus, different measuring time point, such as before sleep or after sleep, may yield different results in OSA patients.

## Conclusion

This study firstly revealed that intermittent hypoxia can upregulate the CCR5 mRNA and protein levels and the chemotaxis of monocytes attracted by RANTES. Also, intermittent hypoxia is shown to promote the adhesion of monocytes to vascular endothelial cells. The p44/42 signaling pathway is confirmed to be involved in the induced CCR5 expression of monocytes by intermittent hypoxia. We also confirmed the upregulation of monocytic CCR5 gene expression in patients with severe OSA. These findings strongly imply a critical role of CCR5 and reveal mechanisms participated in enhanced monocyte adhesion and chemotaxis under intermittent hypoxia. Therefore, the suppression of intermittent hypoxia-induced CCR5 expression or to inhibit the CCR5 function by antagonists could be a reasonable method to avoid the progression of atherosclerosis in OSA patients.
